# Consumer attitudes toward aging skin during the COVID-19 pandemic

**DOI:** 10.1097/JW9.0000000000000095

**Published:** 2023-07-14

**Authors:** Lynna J. Yang, Justin Knoll, Roopal V. Kundu

**Affiliations:** a Northwestern University Feinberg School of Medicine, Chicago, Illinois

**Keywords:** aging, body image, cosmetic dermatology, pandemic, video conferencing

## Abstract

**Background:**

The impact of the novel coronavirus disease (COVID-19) pandemic on consumer attitudes toward their skin has not been well characterized.

**Objective:**

This study investigated how consumers’ attitudes toward their skin changed during the COVID-19 pandemic.

**Methods:**

A cross-sectional survey was conducted using REDCap. A total of 1,434 participants were recruited and consented to participate online through ResearchMatch. The survey gathered demographic information and assessed participants’ attitudes toward their skin using a Likert scale. An ordered logistic regression analysis was performed.

**Results:**

Nearly one-third of participants felt unhappy with their skin. Forty four percent feel less happy about their skin compared with 5 years earlier. The top skin concerns were eye puffiness (86.5%), loose skin (85.1%), uneven tone (84.9%), uneven texture (83.5%), and dry skin (81.4%). Video conferencing (31%), wearing masks (23%), and increased stress (21%) during the COVID-19 pandemic affected how participants felt about their skin. Compared with men, women were 1.6 to 1.8 times (*P* < .01) more likely to “strongly agree” that all 3 pandemic-related factors—video conferencing, wearing masks, and increased stress—affected how they felt about their skin. Younger age groups were 1.5 to 2.8 times (*P* < .01) more likely to answer in the top category for all 3 pandemic-related factors compared with the oldest age group.

**Limitations:**

Recruitment of participants was limited to English-speaking adults aged 18 years or older who were registered on ResearchMatch, which underrepresents minority populations. Further studies should be conducted to elucidate how the pandemic affected perceptions of skin.

**Conclusion:**

Skin aging is a significant concern among adults of all ages. The COVID-19 pandemic has exacerbated skin concerns. Women and young adults are more likely to be affected by the COVID-19 pandemic in their attitudes toward their skin.

**What is known about this subject in regard to women and their families?**

- Many women and their families are consumers of antiaging skin care, a rapidly growing market.
- Video conferencing during the coronavirus disease (COVID-19) pandemic has resulted in an increase in the number of patients seeking dermatologist consultations for skin concerns directly related to use of video conferencing platforms.
- There is concern for a new phenomenon called “Zoom dysmorphia,” in which the increased use of video conferencing platforms could be contributing to body dysmorphic disorder.
- However, consumers’ attitudes toward aging skin, and how the COVID-19 pandemic has affected these attitudes, have not been well characterized.

**What is new from this article as messages for women and their families?**

- Aging skin is a significant and growing concern for many consumers.
- The top age-related facial skin concerns include eye puffiness, loose skin, uneven tone, uneven texture, and dry skin.
- Video conferencing, mask wearing, and increased stress during the COVID-19 pandemic have exacerbated skin concerns for many consumers.
- Women and younger adults are particularly vulnerable to the effects of the COVID-19 pandemic.

## Introduction

Antiaging skin care is a major industry. The global market for antiaging products was worth $62.6 billion in 2021 and is projected to reach around $92 billion by 2027,^1^ suggesting that aging skin is a growing priority for consumers. Although studies have sought to characterize age-related skin changes, there is little understanding of consumers’ knowledge, attitudes, and behavior toward aging skin.

Furthermore, the effects of the global pandemic on consumers’ attitudes toward their skin have not been well characterized. One study found that dermatologists across the country have noticed an increase in the number of patients seeking consultations specifically because they feel dissatisfied with how their skin looks on video conferencing platforms.^2^ Other studies have reported that video conferencing platforms can distort the image of one’s face, which may contribute to consumers’ dissatisfaction with their physical appearance.^3^ Studies have termed this “Zoom dysmorphia,” reminiscent of the “Snapchat dysmorphia,” which raised concerns for body dysmorphic disorder (BDD).^4^ Given these recent findings, a deeper investigation into how the coronavirus disease (COVID-19) pandemic has affected consumers’ attitudes toward their skin was conducted.

## Methods

This study was exempt by the Northwestern University IRB (STU00215051). A cross-sectional, online survey was administered through REDCap between July 5, 2021 and August 31, 2021. Eligible participants (English-speaking participants aged 18 years or older) were recruited and consented online through ResearchMatch, a national free and secure online tool with over 150,000 volunteers in its registry. The survey queried participants about their attitudes toward their skin using a Likert scale (Supplementary Figure S1, http://links.lww.com/IJWD/A20). An ordered logistic regression analysis was performed using STATA to calculate the odds ratio of a certain demographic group answering in the top category (“strongly agree”) relative to all other categories.

## Results

In total, 1,434 participants responded to the survey. Demographic information is summarized in Table 1.

**Table 1:** Demographic information

Variable		n (%)
Age, y	18-30	216 (15.1)
	31-50	408 (28.5)
	51+	810 (56.5)
Gender	Women	1125 (78.5)
	Men	272 (19.0)
	Nonbinary	37 (2.6)
Race/ethnicity	Asian	83 (5.8)
	Black or African American	166 (11.6)
	Hispanic or Latinx	70 (4.9)
	Native American or indigenous	27 (1.9)
	White	1014 (70.7)
	Other	70 (4.9)
Marital status	Single	420 (29.3)
	Married or engaged	708 (49.4)
	Divorced	210 (14.6)
	Widowed	68 (4.7)
	Other	28 (2.0)
Annual household income	$0-24,999	145 (10.1)
	$25,000-49,999	253 (17.6)
	$50,000-74,999	294 (20.5)
	$75,000-99,999	259 (18.1)
	$100,000-199,999	363 (25.3)
	$200,000+	120 (8.4)

y, years.

**Fig. 1.**
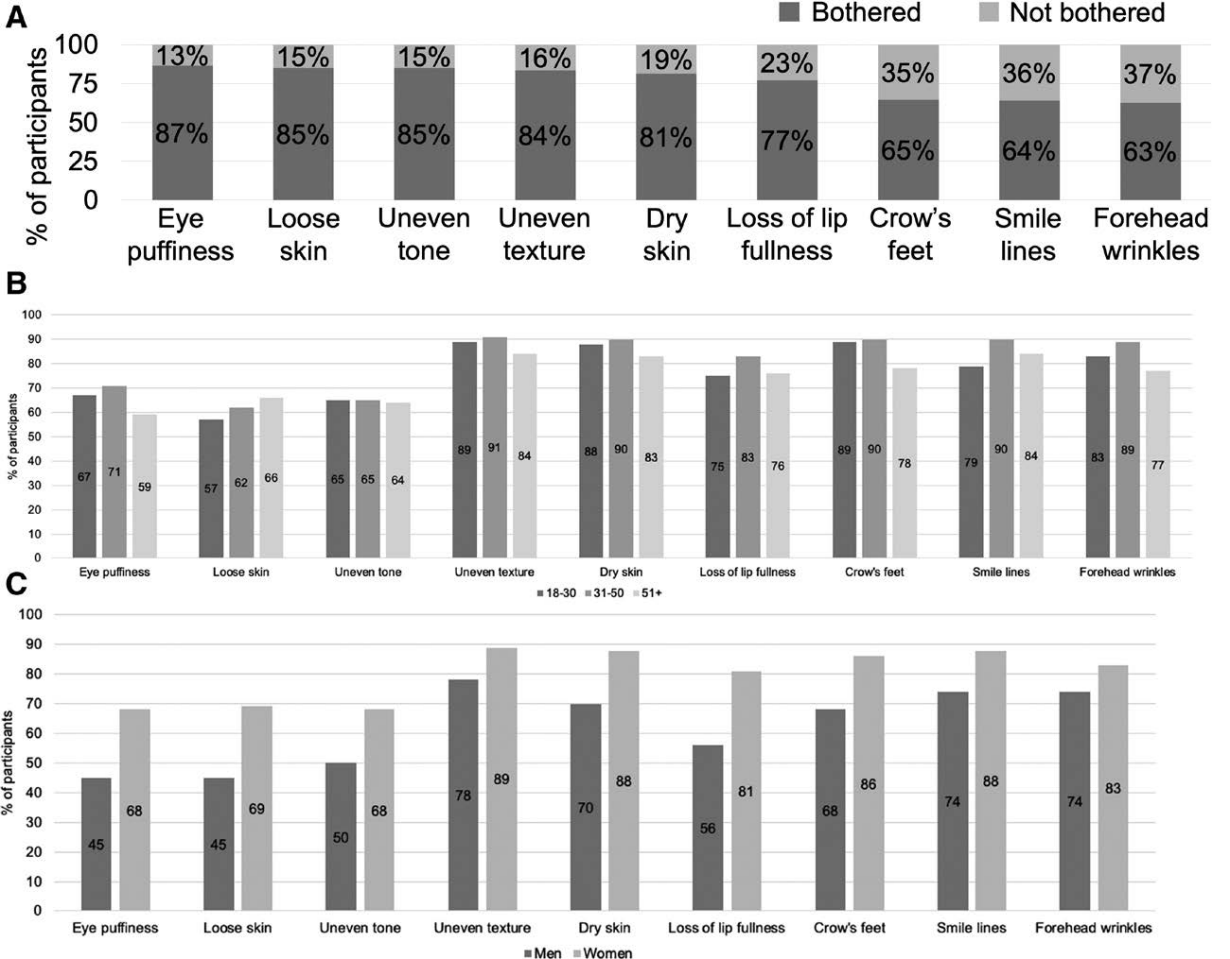
(A) Attitudes toward age-related facial features. Participants were asked whether they were bothered by these age-related skin features. The top 5 facial skin concerns in order were eye puffiness, loose skin, uneven tone, uneven texture, and dry skin. (B) Attitudes toward age-related facial features by age group. The percentages of participants who responded that they were bothered by each age-related skin feature are shown by age group (18-30, 31-50, and 50+ years). (C) Attitudes toward age-related facial features by gender. The percentages of participants who responded that they were bothered by each age-related skin feature are shown by gender.

In the survey, 31% (447/1,434) of the participants reported feeling unhappy, dissatisfied, or unconfident about their skin. Forty-four percent (635/1,434) felt more unhappy, dissatisfied, or unconfident about their skin now compared with 5 years ago. Participants with specific age-related skin features were queried regarding whether they were bothered by or wished to remove these skin features. The top 5 facial skin concerns cited by the participants were eye puffiness (86.5%; 833/963), loose skin (85.1%; 728/857), uneven tone (84.9%; 959/1,129), uneven texture (83.5%; 795/952), and dry skin (81.4%; 796/978) (Fig. 1). However, even lower concerns, including loss of lip fullness, crow’s feet, smile lines, and forehead wrinkles, were bothersome for over 60% of the participants.

Participants were then asked whether their feelings toward their skin were affected by the pandemic, specifically: (1) the increased use of video conferencing platforms such as Zoom; (2) wearing masks; and (3) the stress, anxiety, or self-isolation induced by the pandemic.

**Fig. 2.**
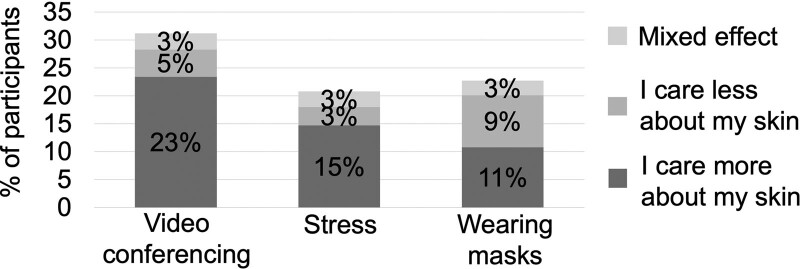
Effect of coronavirus disease (COVID-19) pandemic-related factors on attitudes toward skin. Participants were asked whether the video conferencing, wearing masks, or increased stress during the COVID-19 pandemic affected their attitudes toward their skin. Of the 31% who responded that video conferencing affected how they felt about their skin, 23% felt that it caused them to care more about their skin. Of the 21% who responded that increased stress during the COVID-19 pandemic affected their attitudes toward their skin, 15% cared more about their skin. Of the 23% who felt that wearing masks affected their attitudes toward their skin, 11% cared more, whereas 9% cared less about their skin.

Of the participants who spent more time video conferencing during the COVID-19 pandemic, 31% (401/1,281) responded that video conferencing affected how they felt about their skin. And 23% (300/1,281) felt that they cared more about their skin, while 5% (64/1,281) cared less because of video conferencing (Fig. 2).

Of the participants who wore masks during the pandemic, 23% (317/1,402) reported that wearing masks affected their attitudes toward their skin and 11% (151/1,402) cared more about their skin, whereas 9% (130/1,402) cared less (Fig. 2).

Of the participants who experienced increased stress, anxiety, or self-isolation due to the COVID-19 pandemic, 21% (277/1,334) felt that stress affected their attitudes toward their skin. Fifteen percent (196/1,334) of the participants cared more about their skin, whereas 3% (44/1,334) cared less (Fig. 2).

An ordered logistic regression analysis was performed to analyze differences among demographic groups in how they rated the statement, “Video conferencing platforms/wearing masks/stress, anxiety, or self-isolation caused by the pandemic affected how I feel about my skin.” Compared with men, women were 1.6 to 1.8 times (*P* < .01) more likely to answer that they “strongly agree” that all 3 pandemic factors—stress, wearing masks, and use of video conferencing platforms—affected how they felt about their skin. The youngest age group (age, 18-30 years) were more likely to “strongly agree” that all 3 pandemic-related factors compared with the oldest age group (age, 51 years and older): 1.6 times (*P* < .01) more likely for video conferencing, 2.1 times (*P* < .01) for pandemic-induced stress, and 2.8 times (*P* < .01) for wearing masks. The middle age group (age, 31-50 years) were also 1.5 to 1.9 times (*P* < .01) more likely to answer in the top category for all pandemic-related factors compared with the oldest age group (age, 51 years and older).

## Discussion

Aging skin is a significant concern for adults of all ages, as evidenced by nearly one-third of the participants responding that they felt unhappy, dissatisfied, or unconfident about their skin. Furthermore, concerns about aging skin have increased over time, as nearly half of the participants felt less happy, satisfied, or confident about their skin now compared with 5 years ago. The COVID-19 pandemic has likely played a role in exacerbating skin concerns. These findings are consistent with those of a recent study in which dermatologists reported that their patients seemed more displeased with their current appearance than in the past and are now seeking consultations for skin concerns caused by the COVID-19 pandemic.^2^ Women and young adults were particularly vulnerable to the effects of the COVID-19 pandemic and were more likely to report that the pandemic affected their attitudes toward their skin.

### Top concerns

The top 5 age-related facial features of concern identified in descending order were eye puffiness, loose skin, uneven tone, uneven texture, and dry skin. Most participants were also bothered by other age-related skin features, including loss of lip fullness, crow’s feet, smile lines, and forehead wrinkles. Consistent with these results, eye puffiness and uneven tone were cited as the top concerns for patients in a study surveying dermatologists.^2^ However, these dermatologists also reported upper face wrinkles as a top concern for patients,^2^ while this study found that upper face wrinkles (forehead wrinkles and crow’s feet) were a lower priority than other age-related skin concerns such as loose skin, uneven texture, and dry skin.

### Effects of mask wearing during the COVID-19 pandemic on attitudes toward skin

Wearing masks during the pandemic has had a mixed effect on consumers’ attitudes toward their skin. Almost as many participants reported caring less about their skin because of masks, as did those who reported caring more. Some participants who cared less about their skin because of mask wearing reported that this was because the masks covered areas of their skin. Others reported caring more about their skin because wearing masks led to increased acne outbreaks. Indeed, several studies have found that long-term mask use is associated with exacerbation of acne, termed “maskne,” and other skin conditions such as rosacea, which negatively impact the quality of life.^5^ A study analyzing Tweets about maskne found that many Twitter users expressed frustration about their maskne,^6^ highlighting the impact of mask-related acne on consumers. Despite the prevalence of mask-induced acne, there is a lack of education and guidance regarding its prevention and management.^5,6^

### Effects of the stress, anxiety, or self-isolation of the COVID-19 pandemic on attitudes toward skin

Nearly a quarter of the participants who experienced increased stress, anxiety, or self-isolation during the COVID-19 pandemic reported that these feelings caused them to care more about their skin. Several factors may contribute to why COVID-related stress causes participants to care more about their skin. First, heightened stress levels during the pandemic may have aggravated stress-related skin and hair conditions. A recent study reported an increased incidence of telogen effluvium, alopecia areata, and seborrheic dermatitis among medical students during the pandemic.^7^ Previous studies have suggested that stress can exacerbate acne.^8^ Second, the stress and anxiety of the pandemic may have manifested in lower self-esteem, causing increased skin concerns. A recent study found that pandemic-related stress is associated with a negative body image.^9^ Another study found that negative self-perceptions of aging were correlated with loneliness and psychological distress.^10^ Finally, some participants may have been performing less skincare during the pandemic, which could have exacerbated skin concerns. One study reported that many participants performed less skincare during the pandemic than they did before the pandemic and that most of these participants felt negatively about this change.^11^

### Effects of video conferencing during the COVID-19 pandemic on attitudes toward skin

The use of video conferencing has significantly increased during the COVID-19 pandemic, with 62% of Americans working from home by 2020.^12^ Approximately a quarter of the participants who used video conferencing platforms reported that video conferencing caused them to care more about their skin. This is consistent with the newly elucidated phenomenon, “Zoom dysmorphia,” in which consumers feel unhappy or self-conscious about their appearance during video conferences.^4^ Studies have reported a recent increase in those seeking facial cosmetic procedures, citing the use of video-conferencing platforms as the primary reason.^13^ Many cosmetic procedures that are rising in popularity during the COVID-19 pandemic specifically target facial features of concern, including botulinum toxin for wrinkles, fillers for loose skin, and chemical peels for uneven tone and texture.^13^

Several underlying factors lead video-conferencing users to care more about their skin. First, video conference users find their face constantly visible on the screen, unlike during in-person meetings where participants normally cannot see their own face,^14^ causing video conferencing users to fixate on facial features they otherwise would not focus on. Second, video conferences also provide a platform for users to directly compare their own appearance side-by-side with others in the meeting.^14^ Third, the camera technology of laptops and other digital devices used for video conferences can distort the appearance, leading to greater dissatisfaction. For example, studies have noted that front-facing cameras cause noses to appear broader and eyes to appear farther apart.^15^ Finally, video conferencing platforms can enhance the user’s appearance. For example, Zoom has feature to “touch up my appearance,” which applies a filter to the image to make the skin appear smoother. One study found that video conference users who engaged in appearance comparisons were more likely to use the touch-up feature, spend more time looking at their face on the screen, and report lower appearance satisfaction,^16^ suggesting that these behaviors may be interrelated and harmful to one’s self-image.

Zoom dysmorphia must be recognized because of its potential association with BDD, which is defined by the DSM-5 criteria as having a “preoccupation with one or more perceived defects or flaws in physical appearance that are not observable or appear slight to others.^17^” Although the exact relationship between Zoom dysmorphia and BDD is unclear, studies have compared video conferencing-related dysmorphia with dysmorphia caused by other social media platforms, such as Snapchat.^4^ These social media platforms have filters that alter facial features, such as smoothening skin, narrowing noses, and enlarging eyes, causing users to feel less satisfied with their natural, unfiltered faces.^4^ Studies have linked the use of social media platforms to lower self-esteem and appearance satisfaction. For example, one study found that Facebook use resulted in a more negative mood and a greater desire to change appearance in participants who engaged in appearance comparisons.^18^ Another study found that selfie-viewing behavior on social media was correlated with greater facial dissatisfaction.^19^ This raises the concern that video conferencing-related dysmorphia could similarly precipitate or exacerbate BDD and other mental health issues. One study found that new appearance-related concerns were correlated with an increase in the Dysmorphia Concern Questionnaire score, a validated tool for the assessment of BDD,^20^ suggesting that video conference users with increasing skin concerns may be at risk of developing BDD. This highlights the need for further research on how video conferencing affects users’ self-perceptions and mental health, especially because the use of video conferences is likely to continue beyond the pandemic.

### Women and young adults’ attitudes toward their skin were more likely to be affected by the COVID-19 pandemic

Compared with men, women were more likely to respond that mask wearing, stress, and video conferencing during the COVID-19 pandemic affected their attitudes toward their skin. A 2008 population-based study of BDD estimated the point prevalence of BDD to be slightly higher in women: 2.5% in women and 2.2% in men.^21^ Furthermore, a recent study examining gender differences in adults diagnosed with BDD found that women had higher distress associated with BDD symptoms and poorer illness insight than men.^22^ This suggests that women are not only more likely to develop BDD but may also be at risk of developing more severe symptoms.

Younger adults were also more likely to agree that their attitudes toward their skin were affected by the COVID-19 pandemic than older adults. One study found that the prevalence of BDD is increasing among adolescents, particularly in female adolescents.^23^ Furthermore, one study found that adolescents with BDD had poorer insight and higher suicide rates than adults with BDD.^24^ Taken together, these findings warrant further studies on the effects of the COVID-19 pandemic on mental health, particularly among women and younger age groups.

### Limitations

One of the major limitations of this study was the recruitment of participants. The inclusion criterion was English-speaking adults aged 18 years or older. This excludes non-English-speaking participants and thus does not fully capture minority populations living in the United States. This also excludes children under 18 years of age, including adolescents who are especially vulnerable to BDD. Furthermore, eligible patients were randomly selected and recruited through ResearchMatch. The volunteer population of the ResearchMatch registry is skewed toward White females, resulting in a greater proportion of participants in this study identifying as White and female.

Further studies should be conducted to elucidate how the pandemic has affected perceptions of skin. While this study found that video conferencing impacted attitudes toward skin, the survey did not ask participants to distinguish the purpose of video conferencing. For example, video conferencing for work or school purposes may have a different effect on appearance perception compared with video conferencing for personal or social reasons. Another area for further exploration is how the pandemic affected consumers’ behavior toward aging skin treatment, and how this change in behavior might affect their attitudes toward their skin. For example, delays in skin treatments, such as facials, injectables, or lasers, during the pandemic may partially explain the heightened skin concerns during the pandemic.

## Conclusions

Aging skin is a significant and growing concern among adults of all ages. This study highlights the numerous ways in which the COVID-19 pandemic has exacerbated concerns regarding skin aging. Women and younger age groups are particularly vulnerable to the effects of the COVID-19 pandemic on their attitudes toward their skin. Further studies are needed to better characterize how mask wearing, stress, and video conferencing during the pandemic have affected consumers’ attitudes and behavior toward aging skin to inform recommendations and outreach to consumers.

## Conflicts of interest

None.

## Funding

None.

## Study approval

N/A.

## Author contributions

LJY: Conceived, designed, and administered the survey study; recruited patients online; analyzed and interpreted the data; and drafted the article. JK: Performed statistical analysis and revised the article. RVK: Assisted with study design, data analysis and interpretation, and critical revision of the article. All authors have read and approved the final article.

## Supplementary data

Supplementary material associated with this article can be found at http://links.lww.com/IJWD/A20.

## Supplementary Material


