# Supplementary material for: Consumer attitudes toward aging skin during the COVID-19 pandemic
Source: Int J Womens Dermatol. 2023 Jul 14;9(3):e095. doi: 10.1097/JW9.0000000000000095 (PMC10348726; doi:10.1097/JW9.0000000000000095)
Supplement: Supplementary file 1 [file jw9-9-e095-s001.pdf]

Survey title: Consumer attitude, knowledge, and behavior towards aging skin survey

1. What is your age? (Enter number)

2. What is your gender identity?
   - Man/Male/Masculine
   - Woman/Female/Feminine
   - Transgender Man/Transman/Female-to-male (FTM)
   - Transgender Woman/Transwoman/Male-to-female (MTF)
   - Genderqueer
   - Cisgender
   - Other (please specify)

3. What is your racial/ethnic identity?
   - Asian
   - Black or African American
   - Hispanic or Latinx
   - Native American or Indigenous
   - Native Hawaiian or Pacific Islander
   - White
   - Other (please specify)

4. What is your marital status?
   - Single
   - Married/engaged
   - Divorced
   - Widowed
   - Other (please specify)

5. What is your annual household income?

- $0 – $24,999
- $25,000 – 49,999
- $50,000 – $74,999
- $75,000 – $99,999
- $100,000 – $199,999
- $200,000 or more

6. How do you feel about your skin?
    - 1 = very unhappy, dissatisfied, or not confident
    - 2 = somewhat unhappy, dissatisfied, or not confident
    - 3 = neutral
    - 4 = somewhat happy, satisfied, or confident
    - 5 = very happy, satisfied or confident

7. How have your feelings about your skin changed over the last 5 years?
    - I feel happier, more satisfied, or more confident about my skin
    - I feel less happy, less satisfied or less confident about my skin
    - I feel the same about my skin

8. For the facial features listed below, please rate each category using the following choices: 0 = I don' t have any, 1 = I have a little, 2 = I have a moderate amount of, 3 = I have a lot of
    - Forehead wrinkles
    - Smile lines / wrinkles around the mouth
    - Crow' s feet / wrinkles around the eyes
    - Puffiness under the eyes
    - Loss of fullness / loose skin
    - Loss of lip fullness
    - Uneven texture (ex: dilated pores)
    - Uneven tone/color (ex: sunspots, freckles, dilated blood vessels)
    - Dry skin

9. Follow-up question if answered 1, 2, or 3 for specific facial features in question #8: How do you feel about each of these facial features? Please rate each feature using the following choices: 0 = I don’t wish I could get rid of this and/or this doesn’t bother me, 1 = I slightly wish I could get rid of this and/or this bothers me slightly, 2 = I moderately wish I could get rid of this and/or this bothers me moderately, 3 = I strongly wish I could get rid of this and/or this bothers me a lot.

10. Please rate the following statement: Wearing masks during the pandemic has affected how I feel about my skin: “Strongly agree / somewhat agree / neutral / somewhat disagree / strongly disagree.”

11. Follow-up question if answered “strongly agree or somewhat agree” to question #10: How has wearing masks during this pandemic affected how you feel about your skin? Check all that apply:
    - “I care more about my skin because I get acne from wearing masks”
    - “I care less about my skin because it’s covered by the masks”
    - “Wearing masks has not affected how I feel about my skin”
    - “I don’t typically wear masks”
    - Other (please specify)
    - None of the above

12. Please rate the following statement: Spending more time at home or in front of a screen during the pandemic has affected how I feel about my skin: “Strongly agree / somewhat agree / neutral / somewhat disagree / strongly disagree.”

13. Follow-up question if "strongly agree or somewhat agree" to question #12: How has spending more time at home or in front of a screen during the pandemic affected how you feel about your skin? Check all that apply:
    - "I care more about my skin because I see my face on my screen more."
    - "I care more about my skin because I spend more time in front of a mirror."
    - "I care less about my skin because I spend less time meeting people in-person."
    - "Spending more time at home or in front of a screen has not affected how I feel about my skin"
    - "The pandemic has not affected how much time I spend at home or in front of a screen."
    - Other (please specify)
    - None of the above

14. Please rate the following statement: The stress, anxiety, or self-isolation of the pandemic has affected how I feel about my skin: "Strongly agree / somewhat agree / neutral / somewhat disagree / strongly disagree."

15. Follow-up question if "strongly agree or somewhat agree" to question #14: How has the stress, anxiety, or self-isolation of the pandemic affected the importance of age-related skin changes to you? Check all that apply:
    - "I care more about my skin because of the stress, anxiety, or self-isolation of the pandemic."
    - "I care less about my skin because of the stress, anxiety, or self-isolation of the pandemic."
    - "The stress, anxiety, or self-isolation of the pandemic has not affected how I feel about my skin."
    - "I have not felt extra stress, anxiety, or self-isolation as a result of the pandemic."

- Other (please specify)
- None of the above
